# 5-Azido-4-benz­yloxy-2-meth­oxy-6-methyl­perhydro­pyran-3-ol

**DOI:** 10.1107/S1600536809028657

**Published:** 2009-07-25

**Authors:** Hoong-Kun Fun, Wan-Sin Loh, Sankappa Rai, Prakash Shetty, Arun M. Isloor

**Affiliations:** a X-ray Crystallography Unit, School of Physics, Universiti Sains Malaysia, 11800 USM, Penang, Malaysia; b Syngene International Ltd, Biocon Park, Plot Nos. 2 & 3, Bommasandra 4th Phase, Jigani Link Rd, Bangalore 560 100, India; c Department of Printing, Manipal Institute of Technology, Manipal 576 104, India; d Department of Chemistry, National Institute of Technology-Karnataka, Surathkal, Mangalore 575 025, India

## Abstract

In the title compound, C_14_H_19_N_3_O_4_, the perhydro­pyran ring adopts a chair conformation. An intra­molecular C—H⋯O hydrogen bond generates an *S*(6) ring motif. In the crystal packing, mol­ecules are linked by O—H⋯O hydrogen bonds, forming infinite chains along [100].

## Related literature

For background to d-perosamine, see: Jacquinet (2006 ▶). For the synthesis of d-perosamine, see: Krishna & Agrawal (2000 ▶). For metabolites, see: Grond *et al.* (2000 ▶). For ring conformations, see: Cremer & Pople (1975 ▶). For hydrogen-bond motifs, see: Bernstein *et al.* (1995 ▶). For bond-length data, see: Allen *et al.* (1987 ▶). For the stability of the temperature controller used for the data collection, see: Cosier & Glazer (1986 ▶).
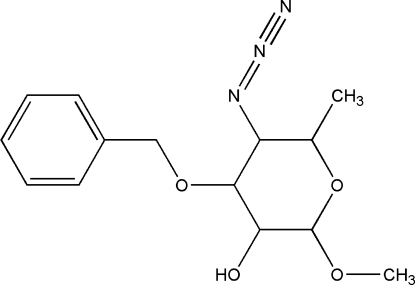


## Experimental

### 

#### Crystal data

C_14_H_19_N_3_O_4_
*M* *_r_* = 293.32
Orthorhombic, 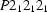

*a* = 4.6662 (2) Å
*b* = 15.3356 (8) Å
*c* = 20.9273 (12) Å
*V* = 1497.54 (13) Å^3^
*Z* = 4
Mo *K*α radiation
μ = 0.10 mm^−1^
*T* = 100 K
0.27 × 0.11 × 0.08 mm

#### Data collection

Bruker SMART APEXII CCD area-detector diffractometer
Absorption correction: multi-scan (**SADABS**; Bruker, 2005 ▶) *T*_min_ = 0.773, *T*_max_ = 0.980
13029 measured reflections
1742 independent reflections
1334 reflections with *I* > 2σ(*I*)
*R*_int_ = 0.096

#### Refinement

*R*[*F* ^2^ > 2σ(*F* ^2^)] = 0.049
*wR*(*F* ^2^) = 0.103
*S* = 1.09
1742 reflections
196 parameters
H atoms treated by a mixture of independent and constrained refinement
Δρ_max_ = 0.23 e Å^−3^
Δρ_min_ = −0.20 e Å^−3^

### 

Data collection: *APEX2* (Bruker, 2005 ▶); cell refinement: *SAINT* (Bruker, 2005 ▶); data reduction: *SAINT*; program(s) used to solve structure: *SHELXTL* (Sheldrick, 2008 ▶); program(s) used to refine structure: *SHELXTL*; molecular graphics: *SHELXTL*; software used to prepare material for publication: *SHELXTL* and *PLATON* (Spek, 2009 ▶).

## Supplementary Material

Crystal structure: contains datablocks global, I. DOI: 10.1107/S1600536809028657/ng2616sup1.cif

Structure factors: contains datablocks I. DOI: 10.1107/S1600536809028657/ng2616Isup2.hkl

Additional supplementary materials:  crystallographic information; 3D view; checkCIF report

## Figures and Tables

**Table 1:** Hydrogen-bond geometry (Å, °)

*D*—H⋯*A*	*D*—H	H⋯*A*	*D*⋯*A*	*D*—H⋯*A*
O2—H1*O*2⋯O2^i^	0.88 (4)	1.91 (3)	2.757 (2)	161 (3)
O2—H1*O*2⋯O3^i^	0.88 (3)	2.49 (3)	3.063 (3)	124 (3)
C7—H7*B*⋯O2	0.97	2.58	3.180 (4)	120

Symmetry code: (i) 
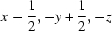
.

**Fig. 1.**
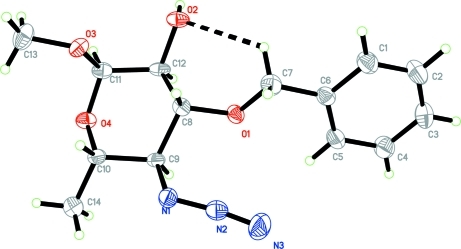
The molecular structure of the title compound, showing 50% probability displacement ellipsoids and the atom numbering scheme. Intramolecular interaction is shown by dashed line.

**Fig. 2.**
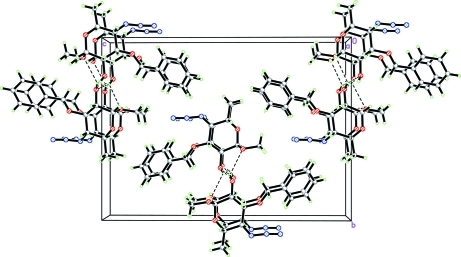
The crystal packing of the title compound, viewed along a axis. Intermolecular hydrogen bonds are shown by dashed lines.

## supplementary crystallographic information

### Comment

4-Amino-4,6-dideoxy-D-mannose (*D*-perosamine) was first discovered in the polyene macrolide antibiotic perimycin and was later recognized to be present in the lipopolysaccharide (LPS) of Vivrio cholera (Jacquinet, 2006). Methyl 3-benzyloxy-4-azido-α-*D*-rhamnopyranoside is an important intermediate in the synthesis of *D*-perosamine (Krishna & Agrawal, 2000). Rhamnopyranosides were detected as metabolites from five different strains of Streptomycetes (Grond *et al.*, 2000).

The bond lengths (Allen *et al.*, 1987) and angles in the molecule (Fig. 1) are within normal ranges. The perhydropyran ring adopts a chair conformation. The puckering parameters (Cremer & Pople, 1975) are Q = 0.540 (3) Å; Θ = 8.0 (3)° and φ = 8.0 (2)°. Intramolecular C7—H7B···O2 hydrogen bonds formed a six-membered ring, producing an *S*(6) ring motif (Bernstein *et al.*, 1995). The dihedral angle formed between the benzene (C1—C6) and perhydropyran (C8—C10/O4/C11/C12) rings is 57.32 (16)°.

In the crystal packing (Fig. 2), the molecules are linked by intermolecular O2—H1O2···O2 and O2—H1O2···O3 hydrogen bonds (Table 1) into an infinite one-dimensional chains along the [100] direction.

### Experimental

To a stirred mixture of methyl 3-benzyloxy-4-methoxysulfonyl-α-*D*-rhamnopyranoside (0.50 g, 1.4 mmol) in DMF (5.0 ml) was added sodium azide (0.18 g, 2.8 mmol). The reaction mixture was stirred further at room temperature for 12 h. TLC (30% EtOAc/hexane, Rf-0.5) analysis showed complete conversion. The reaction mixture was concentrated under vacuum and the residue was purified by column chromatography using 25% ethylacetate in petroleum ether to get pure product as colourless crystals (yield: 300.0 mg, 71%, *M.p.* 376–378 K).

### Refinement

Atom H1O2 was located in a difference map and was refined freely. Other H atoms were positioned geometrically [C—H = 0.93 to 0.98 Å] and was refined using a riding model, with *U*_iso_(H) = 1.2 or 1.5 *U*_eq_(C). A rotating-group model was applied for the methyl groups. In the absence of significant anomalous dispersion, 1187 Friedel pairs were merged for the final refinement.

### Crystal data

**Table:**

C_14_H_19_N_3_O_4_	*F*(000) = 624
*M**_r_* = 293.32	*D*_x_ = 1.301 Mg m^−^^3^
Orthorhombic, *P*2_1_2_1_2_1_	Mo *K*α radiation, λ = 0.71073 Å
Hall symbol: P 2ac 2ab	Cell parameters from 1880 reflections
*a* = 4.6662 (2) Å	θ = 2.4–29.9°
*b* = 15.3356 (8) Å	µ = 0.10 mm^−^^1^
*c* = 20.9273 (12) Å	*T* = 100 K
*V* = 1497.54 (13) Å^3^	Block, colourless
*Z* = 4	0.27 × 0.11 × 0.08 mm

### Data collection

**Table:**

Bruker SMART APEXII CCD area-detector diffractometer	1742 independent reflections
Radiation source: fine-focus sealed tube	1334 reflections with *I* > 2σ(*I*)
graphite	*R*_int_ = 0.096
φ and ω scans	θ_max_ = 26.0°, θ_min_ = 2.0°
Absorption correction: multi-scan (*SADABS*; Bruker, 2005)	*h* = −5→5
*T*_min_ = 0.773, *T*_max_ = 0.980	*k* = −18→17
13029 measured reflections	*l* = −25→25

### Refinement

**Table:**

Refinement on *F*^2^	Primary atom site location: structure-invariant direct methods
Least-squares matrix: full	Secondary atom site location: difference Fourier map
*R*[*F*^2^ > 2σ(*F*^2^)] = 0.049	Hydrogen site location: inferred from neighbouring sites
*wR*(*F*^2^) = 0.103	H atoms treated by a mixture of independent and constrained refinement
*S* = 1.09	*w* = 1/[σ^2^(*F*_o_^2^) + (0.0499*P*)^2^] where *P* = (*F*_o_^2^ + 2*F*_c_^2^)/3
1742 reflections	(Δ/σ)_max_ < 0.001
196 parameters	Δρ_max_ = 0.23 e Å^−^^3^
0 restraints	Δρ_min_ = −0.20 e Å^−^^3^

### Special details

**Table:**

Experimental. The crystal was placed in the cold stream of an Oxford Cyrosystems Cobra open-flow nitrogen cryostat (Cosier & Glazer, 1986) operating at 100.0 (1) K.
Geometry. All e.s.d.'s (except the e.s.d. in the dihedral angle between two l.s. planes) are estimated using the full covariance matrix. The cell e.s.d.'s are taken into account individually in the estimation of e.s.d.'s in distances, angles and torsion angles; correlations between e.s.d.'s in cell parameters are only used when they are defined by crystal symmetry. An approximate (isotropic) treatment of cell e.s.d.'s is used for estimating e.s.d.'s involving l.s. planes.
Refinement. Refinement of *F*^2^ against ALL reflections. The weighted *R*-factor *wR* and goodness of fit *S* are based on *F*^2^, conventional *R*-factors *R* are based on *F*, with *F* set to zero for negative *F*^2^. The threshold expression of *F*^2^ > σ(*F*^2^) is used only for calculating *R*-factors(gt) *etc*. and is not relevant to the choice of reflections for refinement. *R*-factors based on *F*^2^ are statistically about twice as large as those based on *F*, and *R*- factors based on ALL data will be even larger.

### Fractional atomic coordinates and isotropic or equivalent isotropic displacement parameters (Å^2^)

**Table:**

	*x*	*y*	*z*	*U*_iso_*/*U*_eq_	
O1	0.0881 (4)	0.40074 (13)	0.13521 (10)	0.0229 (5)	
O2	0.0911 (5)	0.28256 (14)	0.02574 (11)	0.0251 (5)	
O3	0.3176 (5)	0.39026 (14)	−0.06657 (9)	0.0236 (5)	
O4	−0.0200 (5)	0.49881 (14)	−0.04434 (10)	0.0250 (5)	
N1	0.3572 (6)	0.56890 (17)	0.10102 (12)	0.0260 (7)	
N2	0.3482 (6)	0.56020 (18)	0.16023 (14)	0.0306 (7)	
N3	0.3680 (9)	0.5600 (2)	0.21422 (15)	0.0537 (11)	
C1	0.2228 (8)	0.2487 (2)	0.26646 (17)	0.0341 (9)	
H1A	0.3517	0.2100	0.2479	0.041*	
C2	0.1203 (8)	0.2318 (2)	0.32682 (17)	0.0389 (9)	
H2A	0.1795	0.1820	0.3485	0.047*	
C3	−0.0706 (8)	0.2888 (3)	0.35523 (17)	0.0379 (9)	
H3A	−0.1405	0.2776	0.3960	0.045*	
C4	−0.1561 (9)	0.3619 (2)	0.32269 (16)	0.0362 (9)	
H4A	−0.2856	0.4003	0.3413	0.043*	
C5	−0.0489 (8)	0.3786 (2)	0.26165 (16)	0.0314 (9)	
H5A	−0.1051	0.4289	0.2402	0.038*	
C6	0.1390 (7)	0.3216 (2)	0.23289 (15)	0.0245 (7)	
C7	0.2586 (8)	0.3380 (2)	0.16733 (16)	0.0309 (9)	
H7A	0.4541	0.3590	0.1708	0.037*	
H7B	0.2607	0.2840	0.1431	0.037*	
C8	0.1803 (7)	0.4219 (2)	0.07207 (14)	0.0209 (7)	
H8A	0.3837	0.4073	0.0675	0.025*	
C9	0.1415 (7)	0.51995 (19)	0.06387 (14)	0.0193 (7)	
H9A	−0.0498	0.5361	0.0791	0.023*	
C10	0.1727 (7)	0.5493 (2)	−0.00515 (14)	0.0215 (7)	
H10A	0.3703	0.5397	−0.0193	0.026*	
C11	0.0416 (7)	0.4086 (2)	−0.04432 (15)	0.0241 (8)	
H11A	−0.0965	0.3794	−0.0723	0.029*	
C12	0.0088 (7)	0.37161 (19)	0.02287 (15)	0.0214 (7)	
H12A	−0.1941	0.3754	0.0347	0.026*	
C13	0.3436 (8)	0.4054 (3)	−0.13393 (15)	0.0361 (9)	
H13A	0.5309	0.3880	−0.1480	0.054*	
H13B	0.3159	0.4662	−0.1427	0.054*	
H13C	0.2012	0.3720	−0.1562	0.054*	
C14	0.0949 (9)	0.6437 (2)	−0.01442 (16)	0.0364 (9)	
H14A	0.1146	0.6588	−0.0587	0.055*	
H14B	0.2203	0.6795	0.0107	0.055*	
H14C	−0.0997	0.6530	−0.0012	0.055*	
H1O2	−0.060 (8)	0.251 (2)	0.0151 (15)	0.028 (10)*	

### Atomic displacement parameters (Å^2^)

**Table:**

	*U*^11^	*U*^22^	*U*^33^	*U*^12^	*U*^13^	*U*^23^
O1	0.0225 (12)	0.0219 (12)	0.0243 (11)	0.0014 (10)	0.0055 (10)	0.0037 (10)
O2	0.0200 (12)	0.0185 (12)	0.0368 (13)	−0.0030 (11)	0.0015 (11)	−0.0055 (10)
O3	0.0222 (11)	0.0283 (13)	0.0205 (11)	0.0038 (11)	−0.0013 (10)	−0.0046 (10)
O4	0.0249 (12)	0.0218 (12)	0.0282 (12)	0.0030 (11)	−0.0041 (10)	−0.0033 (10)
N1	0.0301 (16)	0.0255 (17)	0.0224 (16)	−0.0079 (14)	0.0009 (14)	−0.0004 (12)
N2	0.0360 (17)	0.0250 (17)	0.0307 (19)	−0.0096 (14)	−0.0032 (15)	−0.0024 (13)
N3	0.084 (3)	0.049 (2)	0.0274 (18)	−0.028 (2)	−0.0075 (19)	−0.0015 (15)
C1	0.036 (2)	0.032 (2)	0.035 (2)	0.0072 (17)	0.0014 (17)	0.0053 (17)
C2	0.044 (2)	0.032 (2)	0.040 (2)	0.003 (2)	0.000 (2)	0.0127 (18)
C3	0.040 (2)	0.045 (2)	0.0295 (19)	−0.002 (2)	0.0026 (18)	0.0106 (18)
C4	0.040 (2)	0.036 (2)	0.0329 (19)	0.0034 (19)	0.0131 (19)	0.0041 (17)
C5	0.038 (2)	0.0257 (19)	0.0300 (19)	0.0002 (18)	0.0039 (18)	0.0061 (16)
C6	0.0246 (18)	0.0220 (18)	0.0267 (17)	−0.0019 (16)	−0.0029 (16)	0.0018 (15)
C7	0.0305 (19)	0.031 (2)	0.0315 (19)	0.0135 (17)	0.0029 (16)	0.0033 (16)
C8	0.0170 (16)	0.0213 (18)	0.0244 (17)	−0.0001 (15)	0.0013 (15)	0.0015 (14)
C9	0.0197 (16)	0.0150 (16)	0.0233 (16)	0.0001 (14)	0.0031 (15)	−0.0037 (13)
C10	0.0238 (17)	0.0185 (17)	0.0224 (16)	−0.0001 (15)	0.0005 (15)	−0.0037 (14)
C11	0.0224 (16)	0.0213 (18)	0.0286 (18)	−0.0019 (15)	−0.0045 (15)	−0.0047 (15)
C12	0.0153 (15)	0.0157 (16)	0.0333 (18)	0.0017 (14)	0.0013 (15)	−0.0062 (14)
C13	0.035 (2)	0.052 (2)	0.0214 (17)	0.004 (2)	−0.0043 (18)	−0.0014 (17)
C14	0.052 (2)	0.028 (2)	0.0291 (19)	0.0020 (19)	0.0024 (19)	0.0017 (16)

### Geometric parameters (Å, °)

**Table:**

O1—C7	1.418 (4)	C5—H5A	0.9300
O1—C8	1.427 (4)	C6—C7	1.502 (4)
O2—C12	1.420 (3)	C7—H7A	0.9700
O2—H1O2	0.88 (4)	C7—H7B	0.9700
O3—C11	1.398 (4)	C8—C12	1.515 (4)
O3—C13	1.434 (4)	C8—C9	1.524 (4)
O4—C11	1.412 (4)	C8—H8A	0.9800
O4—C10	1.443 (4)	C9—C10	1.520 (4)
N1—N2	1.247 (4)	C9—H9A	0.9800
N1—C9	1.477 (4)	C10—C14	1.505 (4)
N2—N3	1.134 (4)	C10—H10A	0.9800
C1—C2	1.375 (5)	C11—C12	1.524 (4)
C1—C6	1.376 (5)	C11—H11A	0.9800
C1—H1A	0.9300	C12—H12A	0.9800
C2—C3	1.382 (5)	C13—H13A	0.9600
C2—H2A	0.9300	C13—H13B	0.9600
C3—C4	1.371 (5)	C13—H13C	0.9600
C3—H3A	0.9300	C14—H14A	0.9600
C4—C5	1.396 (5)	C14—H14B	0.9600
C4—H4A	0.9300	C14—H14C	0.9600
C5—C6	1.378 (5)		
			
C7—O1—C8	115.1 (2)	N1—C9—C10	106.5 (2)
C12—O2—H1O2	107 (2)	N1—C9—C8	111.2 (3)
C11—O3—C13	111.9 (2)	C10—C9—C8	112.8 (2)
C11—O4—C10	113.5 (2)	N1—C9—H9A	108.7
N2—N1—C9	116.5 (3)	C10—C9—H9A	108.7
N3—N2—N1	171.1 (4)	C8—C9—H9A	108.7
C2—C1—C6	121.5 (3)	O4—C10—C14	107.0 (3)
C2—C1—H1A	119.2	O4—C10—C9	108.8 (2)
C6—C1—H1A	119.2	C14—C10—C9	112.6 (3)
C1—C2—C3	120.0 (3)	O4—C10—H10A	109.5
C1—C2—H2A	120.0	C14—C10—H10A	109.5
C3—C2—H2A	120.0	C9—C10—H10A	109.5
C4—C3—C2	119.4 (3)	O3—C11—O4	112.6 (3)
C4—C3—H3A	120.3	O3—C11—C12	109.0 (3)
C2—C3—H3A	120.3	O4—C11—C12	110.2 (3)
C3—C4—C5	120.1 (3)	O3—C11—H11A	108.3
C3—C4—H4A	120.0	O4—C11—H11A	108.3
C5—C4—H4A	120.0	C12—C11—H11A	108.3
C6—C5—C4	120.7 (3)	O2—C12—C8	108.5 (2)
C6—C5—H5A	119.6	O2—C12—C11	111.7 (2)
C4—C5—H5A	119.6	C8—C12—C11	112.6 (3)
C1—C6—C5	118.3 (3)	O2—C12—H12A	107.9
C1—C6—C7	119.8 (3)	C8—C12—H12A	107.9
C5—C6—C7	121.9 (3)	C11—C12—H12A	107.9
O1—C7—C6	109.8 (3)	O3—C13—H13A	109.5
O1—C7—H7A	109.7	O3—C13—H13B	109.5
C6—C7—H7A	109.7	H13A—C13—H13B	109.5
O1—C7—H7B	109.7	O3—C13—H13C	109.5
C6—C7—H7B	109.7	H13A—C13—H13C	109.5
H7A—C7—H7B	108.2	H13B—C13—H13C	109.5
O1—C8—C12	110.8 (2)	C10—C14—H14A	109.5
O1—C8—C9	107.0 (2)	C10—C14—H14B	109.5
C12—C8—C9	111.3 (3)	H14A—C14—H14B	109.5
O1—C8—H8A	109.2	C10—C14—H14C	109.5
C12—C8—H8A	109.2	H14A—C14—H14C	109.5
C9—C8—H8A	109.2	H14B—C14—H14C	109.5
			
C9—N1—N2—N3	−177 (2)	C12—C8—C9—C10	−47.1 (4)
C6—C1—C2—C3	−0.2 (6)	C11—O4—C10—C14	175.5 (3)
C1—C2—C3—C4	0.0 (6)	C11—O4—C10—C9	−62.7 (3)
C2—C3—C4—C5	−0.5 (6)	N1—C9—C10—O4	176.0 (2)
C3—C4—C5—C6	1.2 (6)	C8—C9—C10—O4	53.7 (3)
C2—C1—C6—C5	0.9 (5)	N1—C9—C10—C14	−65.6 (3)
C2—C1—C6—C7	179.4 (3)	C8—C9—C10—C14	172.2 (3)
C4—C5—C6—C1	−1.4 (5)	C13—O3—C11—O4	−70.6 (3)
C4—C5—C6—C7	−179.8 (3)	C13—O3—C11—C12	166.9 (2)
C8—O1—C7—C6	−179.9 (3)	C10—O4—C11—O3	−59.5 (3)
C1—C6—C7—O1	163.7 (3)	C10—O4—C11—C12	62.4 (3)
C5—C6—C7—O1	−17.8 (5)	O1—C8—C12—O2	−71.0 (3)
C7—O1—C8—C12	98.3 (3)	C9—C8—C12—O2	170.1 (3)
C7—O1—C8—C9	−140.2 (3)	O1—C8—C12—C11	164.8 (2)
N2—N1—C9—C10	173.4 (3)	C9—C8—C12—C11	45.9 (3)
N2—N1—C9—C8	−63.3 (4)	O3—C11—C12—O2	−51.3 (3)
O1—C8—C9—N1	72.2 (3)	O4—C11—C12—O2	−175.3 (2)
C12—C8—C9—N1	−166.7 (2)	O3—C11—C12—C8	71.1 (3)
O1—C8—C9—C10	−168.2 (2)	O4—C11—C12—C8	−52.9 (3)

### Hydrogen-bond geometry (Å, °)

**Table:**

*D*—H···*A*	*D*—H	H···*A*	*D*···*A*	*D*—H···*A*
O2—H1O2···O2^i^	0.88 (4)	1.91 (3)	2.757 (2)	161 (3)
O2—H1O2···O3^i^	0.88 (3)	2.49 (3)	3.063 (3)	124 (3)
C7—H7B···O2	0.97	2.58	3.180 (4)	120

Symmetry codes: (i) *x*−1/2, −*y*+1/2, −*z*.
